# Gender Differences in Psychological Impact of the Confinement During the COVID-19 Outbreak in Spain: A Longitudinal Study

**DOI:** 10.3389/fpsyg.2021.682860

**Published:** 2021-06-24

**Authors:** Javier Fenollar-Cortés, Óliver Jiménez, Antonio Ruiz-García, Davinia M. Resurrección

**Affiliations:** ^1^Department of Psychology, Universidad Loyola Andalucía, Dos Hermanas, Spain; ^2^Department of Personality, Assessment and Psychological Treatment, Universidad de Málaga, Málaga, Spain; ^3^Department of Psychology, Universidad de Córdoba, Córdoba, Spain

**Keywords:** COVID-19, longitudinal study, psychological impact, gender differences, coronavirus—COVID-19

## Abstract

The rapid spread of the coronavirus disease 2019 (COVID-19) has led the authorities to establish compulsory confinement for most of the Spanish population from March to May 2020. Severe isolation combined with the uncertainty and fear associated with the public health crisis can have a psychological impact on the general population. The aim of the current study was to compare possible gender differences in mental health and psychological measures throughout the confinement. One hundred and sixty-four Spanish participants (75% female; M_age_ = 39.8; SD = 13.5) completed the surveys at the beginning, middle, and end of the forced confinement. The psychological variables were associated with depressive, anxiety, stress, and intrusive/avoidance symptoms, as well as a total score for overall mental health, and a positive/negative affect measure. The results showed that although females had significantly higher scores than males in almost all measures at the beginning of the confinement, the gender differences were quickly vanishing away over time. In fact, intra-group analysis showed that while the female group significantly improved their results on most psychological measures, the male group improved on only one single measure. In summary, the results showed that although the female group started the confinement with higher levels of negative emotions (particularly symptoms of stress and avoidance) than the male group, these differences were significantly reduced in the first few weeks due to the overall improvement in the results of the female group.

## Introduction

**Figure 1:**
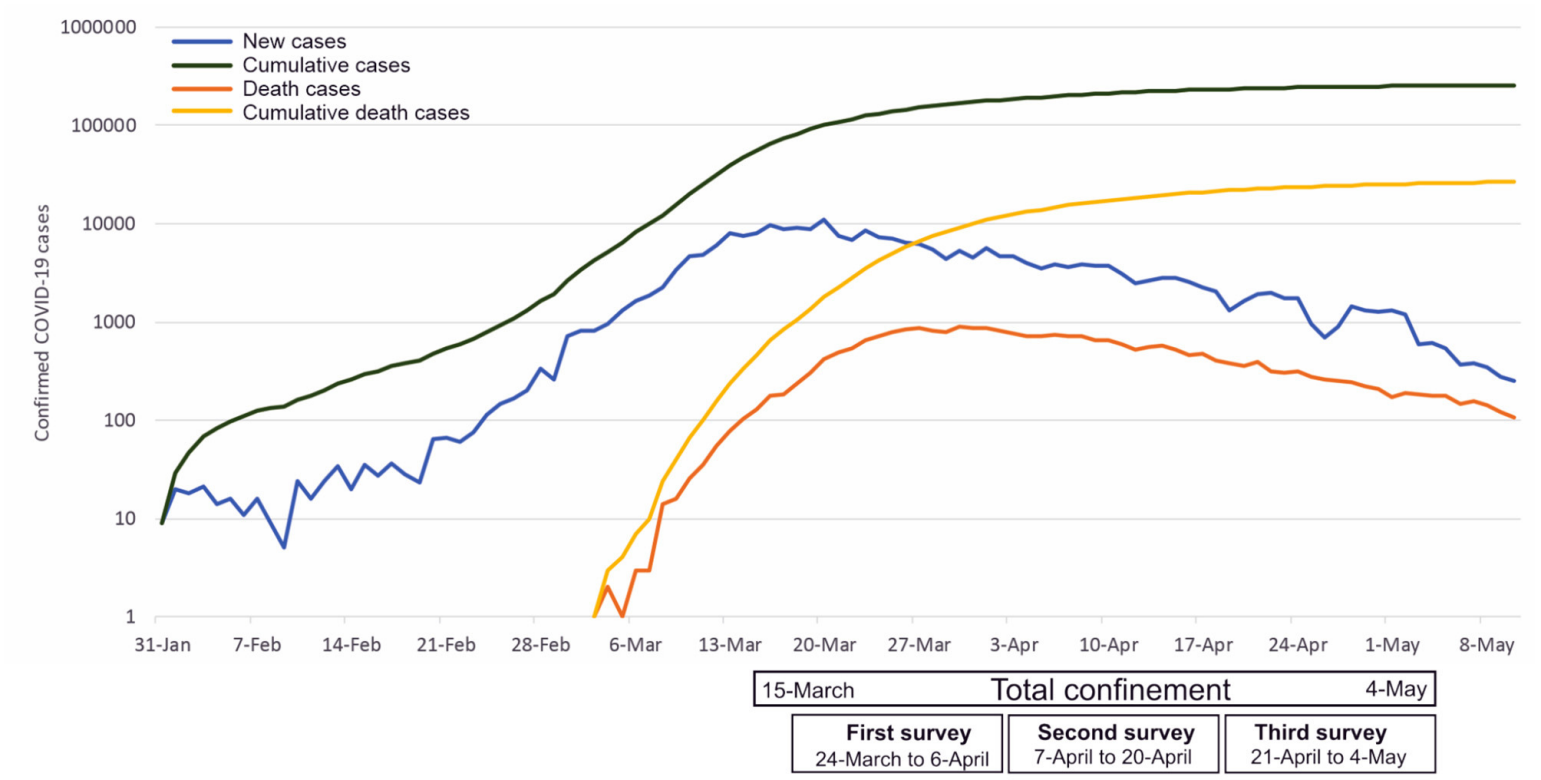
National epidemic trend of 2020 covid disease (COVID-19) outbreak in Spain.

On 30 January 2020, the World Health Organization (WHO) declared the emergence of the novel coronavirus emergence (Eurosurveillance Editorial Team, 2020), provoking pneumonia of unknown etiology in Wuhan, China. This novel coronavirus is named 2019-nCoV or SARS-Cov-2 also known as COVID-19 (Wu et al., 2020). In the past two decades, SARS-Cov-2 is the third coronavirus outbreak (Guarner, 2020). Since the first case registered in December 2019, there have been more than 121 million human infections worldwide with more than two million deaths, overcoming the number of infections in the SARS outbreak in 2003 (WHO, 2021). COVID-19 is considered highly pathogenic and has quickly spread globally due in part to its fast reproducibility estimated in ranges from 2.24 (95% CI: 1.95–2.55) to 5.71 (95% CI: 4.24–7.54) (Zhao et al., 2020). Namely, a person can infect ~2 to 4 people (Palacios et al., 2020). In addition to this high transmissibility, the incubation period is about 6.4 days of average (ranging from 2.1 to 11.1 days) (Zhao et al., 2020). Therefore, the WHO declared COVID-19 a public health emergency of international concern (Mahase, 2020). Because of the rapid spread of COVID disease and following WHO recommendations about, in March 2020 the Spanish authorities established a compulsory confinement in the country (Agencia Estatal BOE 463/2020). This confinement took place from March 14 to June 21, 2020 and it included quarantine measures such as the cessation of all non-essential activities, activities were limited to basic needs such as buying supplies or medication, attending health centers or financial institutions, and caring for vulnerable people. At the time of writing, March 2021, global coronavirus surpassed 120 million cases, with more than two million deaths (WHO, 2021). In Europe, over 5 million people have been infected, with almost 233,692 deaths, and Spain is the fourth country with most cases in Europe, with more than 3 million cases, and the sixth country with most deaths (WHO, 2021). Spain was one of the countries particularly affected by the covid-19 pandemic. Strict confinement of the population allowed the COVID-19 infection and death curves to fall (see Figure 1).

Beyond the medical risk, the COVID pandemic has a psychological impact on the mental health of the population. The initial outbreak provoked media information overload, panic buying of necessity goods, feelings of social isolation and symptoms related to the disruption of the everyday plans (Ho et al., 2020). At the initial phase of the lockdown, diverse psychiatric comorbidities appeared, including persistent depression, anxiety, and panic attacks (Courtet et al., 2020). Following a metanalysis that included 17 studies, the prevalence of stress, anxiety and depression in the general population was 29.6, 31.9, and 33.7%, respectively (Salari et al., 2020). In this sense, a systematic review found that the prevalence of depressive symptoms ranged from 14.6 to 48.3%, and for the anxiety symptoms from 6.33 to 50.9% (Xiong et al., 2020). This symptomatology may persist for several months, especially those symptoms related to posttraumatic stress (Courtet et al., 2020). One of the results highlighted in recent studies is the gender differences in the psychological impact of COVID pandemic. In this sense, the female gender is associated with a greater vulnerability to stress, to posttraumatic stress disorder (PTSD), and to depression (Salari et al., 2020; Xiong et al., 2020), and showing a higher prevalence of anxiety and depression (Salari et al., 2020). These gender differences are similar to those findings before the pandemic situation where women showed higher psychological distress than men (Matud et al., 2015; Auerbach et al., 2018). Taken together these results, it is important to attend to the needs of the general population who might need emotional support. The literature pointed that being women is a risk factor for showing worse mental health status during the pandemic (Pappa et al., 2020; Parrado-González and León-Jariego, 2020).

Specifically in Spain, several studies have found that compared to men, women presented higher emotional discomfort, worse mental health status (Parrado-González and León-Jariego, 2020), worse psychological responses to the pandemic (Justo-Alonso et al., 2020), and higher emotional vulnerability to the effects of the lockdown period (Sandín et al., 2020).

Thus, and taking into account previous literature that highlighted the relevance of analyzing the psychological effects during the lockdown both short and long term (Brooks et al., 2020; Wang et al., 2020a,b; Zhang et al., 2020) and following the proposal made by several studies (Castellanos-Torres et al., 2020; Justo-Alonso et al., 2020; Parrado-González and León-Jariego, 2020; Ruiz-Cantero, 2020; Salari et al., 2020; Sandín et al., 2020; Xiong et al., 2020) and the Gender and COVID-19 Working Group (Wenham et al., 2020), there is a need to consider the gender effects of the COVID outbreak. In addition, Spanish studies have already suggested the need for longitudinal data at a prospective level (González-Sanguino et al., 2020). Thus, the main objective of the present study was to analyse the differences between genders in the longitudinal psychological impact of the COVID-19 outbreak in Spain, from March 24 to May 4, 2020.

## Methods

### Study Design

This longitudinal study was launched to the participants for 6 weeks, from March 24th until the end of the lockdown, on May 4th.

### Participants

**Figure 2:**
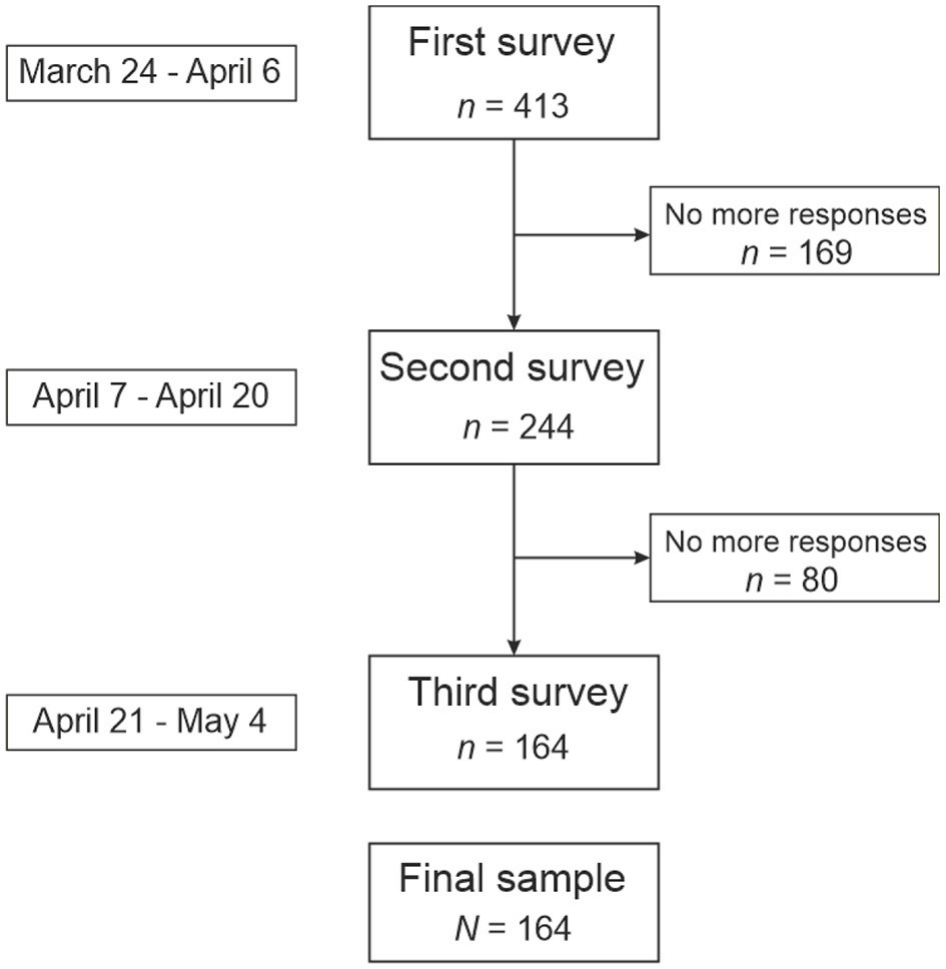
Flow chart indicating the sample size and missing data throughout the collecting data process.

A convenience sample participated in the study. All participants were informed of the objectives and procedure of the study. The free, prior and informed consent was a necessary condition to collaborate in the study. The Commission on Ethics in Research of the Universidad Loyola Andalucia approved the protocol for the study. Inclusion criteria were (a) being older than 18 years old, and (b) be resident in Spain. The final group consisted of 164 participants, with ages ranging from 18 to 77 years, residents in Spain (see Figure 2).

### Instruments

The following questionnaires were included in a battery created using Google Forms and sent out through mail. The outcomes measures for the study assess symptoms related to depression, anxiety, and stress, as well as affect value, subject distress, and psychological well-being. We now describe the scales used to select these outcome measures along with the predictors. Also, sociodemographic data were collected as gender, sex, medical status, education level, living conditions, marital status, and employment status.

- The Positive and Negative Affect Schedule (PANAS scales; Watson et al., 1988; Spanish validation by Sandín et al., 1999).

PANAS is a 20-item self-report measure assessing the frequency of experiencing positive affect and negative affect subscales. Each subscale contains 10 items rated from 1 (*Very slightly or not at all*) to 5 (*Extremely*). Total score ranges from 10 to 50 by subscale, with higher scores representing higher levels of positive affect and lower scores representing lower levels of negative affect for Positive and Negative Affect subscales, respectively. MacDonald's ω was 0.89 (first survey), 0.94 (second survey), and 0.95 (third survey) for the Positive Affect subscale; and 0.88 (first survey), 0.89 (second survey), and 0.93 (third survey) for the Negative Affect subscale.

- Depression, Anxiety and Stress Scale-21 (DASS-21) (Lovibond and Lovibond, 1995; Spanish adaption by Daza et al., 2002).

DASS-21 is a 21-item self-report scale with depression, anxiety, and stress scales. Each item was rated on a 4-point frequency of occurrence scale for the past week (0 = *Did not apply to me at all*, 1 = *Applied to me to some degree, or some of the time*, 2 = *Applied to me to a considerable degree or a good part of the time*, 3 = *Applied to me very much or most of the time*). Each of the three DASS-21 scales contains 7 items with similar content. Total scores for depression, anxiety, and stress are calculated by summing the scores for the relevant items. MacDonald's ω was 0.87 (first survey) and 0.90 (second and third survey) for the Depression scale; 0.88 (first survey) and 0.90 (second and third survey) for the Anxiety scale; and, finally, 0.89 (first survey), 0.90 (third survey), and 0.94 (third survey) for Stress scale

- Impact of Event Scale (IES) (Horowitz et al., 1979; Spanish adaption by Báguena et al., 2001).

The IES is a self-report scale to measure current subjective distress related to a specific event. The scale consists of 15 items, seven of which measure intrusive symptoms, and eight items measure avoidance symptoms. Each item was rated on a 4-point frequency of occurrence scale for the past 7 days (0 = *Not at all*, 1 = *Rarely*, 3 = *Sometimes*, 5 = *Often*), with higher scores representing higher levels of intrusive and avoidance symptoms. Total scores for intrusive symptoms and avoidance symptoms are calculated by summing the scores for the relevant items. MacDonald's ω was 0.81 (first survey), 0.83 (second survey), and 0.88 (third survey) for the Intrusive symptoms scale; and, 0.82 (first survey) 0.83 (second survey), and 0.88 (third survey) for the Avoidance symptoms scale.

- Mental Health Inventory (MHI-5) (Berwick et al., 1901; Spanish adaption by Vilagut et al., 2005).

The MHI-5 is a brief version that includes items on psychological well-being. Each item asked respondents to rate on a six-point frequency or intensity scale how they had been feeling during the previous 4 weeks (from “*All the time*” to “*None of the time*”; the third and fifth items have reverse scoring). The MHI-5 total score is transformed into a variable ranging from 0 to 100, where a score of 100 represents optimal mental health. MacDonald's ω was 0.82, 0.85, and 0.89 (for the first, second, and third surveys, respectively).

### Procedure

Given the country's health situation and the general confinement of the population, the sociodemographic and clinical measures were collected using Google Forms, using a snowball sample method through social media such as Twitter, Facebook, or Whatsapp. Participation was voluntary with no incentive provided. Data collection was carried out weekly from the beginning to the end of the confinement. In the first contact, the purpose and methodology of the study were reported, informed consent was requested, clinical scales were applied, and sociodemographic data were collected. At the end of the first survey, a code was assigned to each participant, which would be the one to be entered in future surveys. Follow-up assessments were administered through a link sent to the email every Monday. Weekly, all the participants who completed the first assessment, received an email with a link for the next one. Successive surveys did not include sociodemographic questions but questions regarding possible changes in the participant's situation. The order of the measures was always the same throughout the surveys.

Only participants who had completed at least one scale in each of the study time intervals were included in the study. That is, from March 24th to April 6th, the first period; from April 7th to April 10th, the second period; from April 21st to May 4th, the third period. Completed surveys with missing data were not included. The first survey was completed by 798 participants. Four of them were excluded because they were minors. Three hundred and eighty one participants did not respond to any surveys again, so they were excluded. Two hundred and forty nine participants did not complete at least one survey at one of the study time intervals, so they were not included. Finally, 164 participants met all the criteria for inclusion, and they were included in the study.

### Analytic Strategy

Given the sample size (*N* = 164), to explore the data distribution, both the Normal Q-Q plot was explored, and the z statistic was calculated for all the psychological outcomes (Kim, 2013). The cut-off point for the z value was ±3.29 (Mayers, 2013). Except for PANAS positive affect scores (z = 0.12 and z = −0.22, first and second surveys, respectively), the rest of the measures were non-normally distributed.

To explore the possible differences between age range groups and the psychological outcomes, Kruskal-Wallis analyses were conducted, and eta squared was calculated as effect size statistic (Tomczak and Tomczak, 2014) (η^2^ =0.001, 0.06, and 0.14, as a small, medium, and large effect, respectively; Cohen, 1988). *Post-hoc* tests using Dunn's test with Bonferroni correction were also conducted. To explore the possible gender differences, the Kruskal-Wallis test (with the age as a covariate) and Mann-Whitney test were conducted, using rank biserial *r* as effect size statistic (*r* = 0.1, 0.3, and 0.5, as a small, medium, and a large effect; Cohen, 1988).

Friedman's tests were carried out to explore the possible within-group differences in each of the psychological measures throughout the confinement, differentiating by gender. Additionally, Conover tests were used for *post-hoc* analysis. Kendall's W statistic was used as an effect size estimation (from 0, indicating no relationship, to 1, indicating a perfect relationship; Tomczak and Tomczak, 2014).

Finally, Cochran's Q test was conducted to explore the possible differences in the answer frequencies for qualitative items at the onset, middle, and ending of the study.

## Results

The majority of the respondents were females (75.0%), not belonging to risk groups (77.4%), not under medical or psychological treatment at the moment of the study (77.4%), living with the family (56.1%), high-educated (69.5% with at least a bachelor's degree), and currently working (62.8%, whether employed or self-employed). The mean age of the sample was 39.8 (*SD* = 13.5; males, *M* = 43.8, *SD* = 15.2; females, *M* = 38.9, *SD* = 12.7; *t* = 2.26, *p* = 0.025, Cohen's *d* = 0.35). Most of the participants were of Spanish nationality (94.5%) and lived in urban areas from 24 Spanish provinces during the COVID-19 confinement. Except for Education level [χ^2^(3, *N* = 164) = 14.52, *p* < 0.01], there were no significant differences by sex for any other sociodemographic variables. More than half of the respondents (59.8%) were quite or very satisfied with the measures adopted by the authorities. According to the age distribution of the sample, three groups were established according to the following age ranges: the first group, from 18 to 33 years; the second group, from 34 to 45 years; and the third group, from 46 to 77 years. A summary of the participants' sociodemographic information is shown in Table 1.

**Table 1:** Sociodemographic characteristics of the total sample (*N* = 164).

	**Total sample *n* (%)**
**Gender**	
Male	41 (25.0)
Female	123 (75.0)
**Age range**	
18–33	60 (36.6)
34–45	53 (32.3)
46–77	51 (31.1)
**Medical status**	
No	127 (77.4)
Medical	24 (14.6)
Psychological	13 (7.6)
**Risk group**	
Yes	37 (22.6)
No	127 (77.4)
**Marital Status**	
Single	48 (29.3
Married	58 (35.4)
Couple	43 (26.2)
Divorced/separated	12 (7.3)
Other	3 (1.8)
**Living**	
With the family	92 (56.1)
With a partner	46 (28.0)
With roommate/s	8 (4.9)
Alone	18 (11.0)
**Education level**	
Up to General Certificate of Education	26 (15.9)
Certificate of Higher Education	24 (14.6)
University Degree	68 (41.5)
Master's Degree	46 (28.0)
**Employment status**	
Employee	55 (33.5)
Self-employed	16 (9.8)
Unemployed	12 (7.3)
Public officer	32 (19.5)
Domestic work	5 (3.0)
Student	26 (15.9)
Retired	8 (4.9)
Other	10 (6.1)

### Data Distribution and the Age as a Covariate

Given the sample size (*N* = 164), to explore the data distribution, both the Normal Q-Q plot was explored, and the z statistic was calculated for all the psychological outcomes (Kim, 2013). The cutoff point for the z value was ±3.29 (Mayers, 2013). Except for PANAS positive affect scores (*z* = 0.12 and *z* = −0.22, first and second surveys, respectively) the rest of the measures were non-normally distributed.

The age and some of the psychological measures were significantly correlated (Table 2). Particularly, the age was moderately negatively correlated with DASS-21 General stress symptoms scores [Spearman's ρ(164) = −0.37, *p* < 0.001, in the second survey, and ρ(164) = −0.25, *p* = 0.001, both the first and the third surveys]. DASS-21 Depressive symptoms scores were also negatively correlated with age [ρ(164) = −0.26 and ρ(164) = −0.28, *p*s < 0.001, the second and the third survey, respectively]. Furthermore, the age was negatively slightly correlated with IES avoidance in all the measurements (ρ*s* = −0.16 to −0.20). The correlations between the age and the psychological measures were higher in the second and third surveys.

**Table 2:** Spearman correlations between the age and the psychological measures.

	**Onset (March 24–April 6)**	**Middle (April 7–April 20)**	**Ending (April 21–May 4)**
**PANAS**			
Positive affect	0.10	0.27^***^	0.22^**^
Negative affect	−0.13	−0.15	−0.04
**MHI5**			
Total score	0.14	0.30^***^	0.21^**^
**DASS-21**			
Depressive symptoms	−0.15	−0.26^***^	−0.28^***^
Anxiety symptoms	−0.15	−0.25^***^	−0.19^**^
General stress symptoms	−0.25^***^	−0.37^***^	−0.25^**^
**IES**			
Intrusive symptoms	−0.07	−0.02	−0.07
Avoidance symptoms	−0.17^**^	−0.20^**^	−0.16^**^

*PANAS, The Positive and Negative Affect Schedule; MHI5, Mental Health Inventory; DASS-21, Depression, Anxiety and Stress Scale; IES, Impact of Event Scale*.

* *p < 0.05*;

** *p < 0.01*;

*** *p < 0.001*.

As age-range groups, there were statistically significant differences in some psychological measures between groups. In all those differences between significant groups, the older group (46 to 77 years) obtained better mean scores than the younger group (18 to 33 years). For example, the general stress symptoms median scores were significantly lower for the 46 to 77 years old group than the 18 to 33 years group in the first [*H*_(2)_ = 8.43, *p* = 0.015, η^2^ = 0.04], second [*H*_(2)_ = 19.3, *p* < 0.001, η^2^ = 0.11], and third [*H*_(2)_ = 9.10, *p* = 0.011, η^2^ = 0.04] surveys. Also, the PANAS positive affect median scores were significantly higher for the 46 to 77 years old group than the 18 to 33 years group in the second [*H*_(2)_ = 12.8, *p* = 0.002, η^2^ = 0.07] and the third [*H*_(2)_ = 8.11, *p* = 0.017, η^2^ = 0.04] surveys. While the DASS-21 Depressive and Anxiety symptoms median scores were significantly higher for the 18 to 33 years group in the second [*H*_(2)_ = 10.9, *p* = 0.004, η^2^ = 0.06, for Depressive symptoms median scores; *H*_(2)_ = 6.53, *p* = 0.038, η^2^ = 0.03, for Anxiety symptoms median scores] and the third [*H*_(2)_ = 10.8, *p* = 0.004, η^2^ = 0.06, for Depressive symptoms median scores; *H*_(2)_ = 6.48, *p* = 0.039, η^2^ = 0.03, for Anxiety symptoms median scores] surveys.

### Comparison Between Male and Female Responder for the Psychological Outcomes Between the First, Second, and Third Survey

As can be seen in Table 3, there were significant differences between males and females' participants for some of the psychological outcomes (particularly, for the first survey). Mann-Whitney test and Kruskal Wallis (age as a covariate) indicated that males had greater MHI-5 Total scores than females in all three surveys, although these differences were decreasing slightly with respect to the size of the effect (from η^2^ = 0.30 to *r* = 0.03). The rest of the differences between males and females were disappearing over time. For example, females had significantly greater General stress symptoms scores (*M* = 6.15, *SD* = 4.87) than males (*M* = 3.00, *SD* = 3.25) in the first survey [*t*_(162)_ = −3.88, *p* < 0.001, *d* = −0.70]. However, that difference was not significant neither in the second survey [*t*_(162)_ = −1.92, *p* = 0.056] nor in the third survey [*t*_(162)_ = −1.73, *p* = 0.085]. That is, the general stress level decreased in females throughout the confinement, while it increased slightly in males. Similarly, the differences for Negative affect (PANAS), Depressive symptoms (DASS-21), and Avoidance symptom scores between male and female participants ceased to be significant after the first survey.

**Table 3:** Comparison between female and male groups on the psychological measures in the first, second, and third surveys.

	**Onset (March 24–April 6)**				**Middle (April 7–April 20)**				**Ending (April 21–May 4)**			
	**Female**	**Male**			**Female**	**Male**			**Female**	**Male**		
**PANAS**			**U**^**b**^**/H**^**a**^	**r/η^2^**			**U**^**b**^**/H**^**a**^	**r/η^2^**			**U**^**b**^**/H**^**a**^	**r/η^2^**
Positive affect	28.2 (8.21)	27.9 (7.51)	2,349^b^	−0.07.	29.3 (9.83)	27.9 (7.90)	0.89^a^	−0.10	29.8 (10.0)	28.0 (8.93)	0.75^a^	−0.09
Negative affect	18.7 (7.14)	16.0 (5.79)	1,964^b^	−0.22^*^	17.4 (7.00)	16.5 (8.18)	2,283^b^	−0.09	16.7 (7.85)	16.3 (8.16)	2,336^b^	−0.07
**MHI5**												
Total score	64.9 (17.3)	74.3 (14.5)	3,329^b^	0.30^**^	67.2 (18.4)	75.7 (16.0)	6.21^a^	0.03^*^	68.1 (20.3)	76.9 (16.6)	5.72^a^	0.03^*^
**DASS-21**												
Depressive symptoms	3.42 (3.93)	1.85 (2.72)	1,796^b^	−0.29^**^	3.44 (4.28)	2.59 (3.46)	−1.44^a^	−0.12	3.14 (4.12)	2.54 (3.47)	0.92^a^	−0.10
Anxiety symptoms	2.61 (3.90)	1.49 (2.44)	2,138^b^	n.s.	2.18 (3.66)	1.46 (3.21)	3.51^a^	0.06	2.33 (4.02)	1.46 (2.84	1.42^a^	−0.12
General stress symptoms	6.15 (4.87)	3.00 (3.25)	15.2^a^	0.09^***^	5.77 (5.03)	4.10 (4.16)	3.73^a^	−0.20	5.42 (5.47)	3.81 (4.12)	2.37^a^	−0.16
**IES**												
Intrusive symptoms	11.3 (7.25)	9.46 (6.87)	2,124^b^	−0.16	9.40 (6.70)	6.56 (6.79)	1,794^b^	−0.29^**^	7.94 (7.71)	6.49 (7.60)	2,157^b^	−0.14
Avoidance symptoms	15.2 (9.16)	10.3 (7.27)	9.26^a^	0.05^**^	12.4 (8.77)	9.68 (7.9)	3.05^a^	0.04	10.6 (9.63)	8.05 (8.12)	2.16^a^	0.05

*PANAS, The Positive and Negative Affect Schedule; MHI5, Mental Health Inventory; DASS-21, Depression, Anxiety and Stress Scale; IES, Impact of Event Scale*.

* *p < 0.05*;

** *p < 0.01*;

*** *p < 0.001*.

a *Kruskal-Wallis H test with age as covariate. η^2^ to effect size estimation*.

b *Mann Whitney U test. rank biserial r to effect size estimation*.

It should be noted that the “intrusive symptoms” score showed some differences with respect to the pattern of the other scores. While there were no significant differences between male and female participants for Intrusive symptoms median scores both in the first (*U* = 2124, *p* = 0.131) and the third (*U* = −2,157, *p* = 0.165) surveys, the differences were significant in the second survey *U* = 1,794, *p* = 0.006, *r* = −29; *Mdn* = 9 and 5 for females and males, respectively). However, even though both groups decreased their intrusive symptoms mean scores throughout the confinement, the decrease was more pronounced in the group of males, particularly between the first and the second survey.

### Within-Group Comparisons by Gender of the Psychological Measures Over Time

Friedman and Conover tests were conducted to compare the effect of confinement on the psychological outcomes, at the onset, middle, and ending of the confinement. Kendall's W statistic was used as an effect size estimation.

There was a significant main effect of the moment of the confinement on PANAS Negative affect median score χ2_(3)_ = 23.9, *p* < 0.001, Kendall's *W* = 0.78. Conover's *post hoc* comparison revealed that for female group, the PANAS negative affect median score in the onset confinement (*Mdn* = 17) was higher than both in the middle (*Mdn* = 15) (*p* = 0.010) and in the ending (*Mdn* = 14) (*p* < 0.001). The median scores for PANAS negative affect were also higher in the middle than in the ending of confinement (*p* = 0.023). For the male group, there were no differences between PANAS Negative affect median scores throughout the confinement (*p* = 0.631).

IES intrusive and avoidance symptoms total scores decreased significantly as time increased both for males and female groups (Table 4). For the female group, the differences were significant between onset and ending confinement, as well as between middle and ending confinement (all differences were *p* < 0.001, both IES intrusive and avoidance symptoms median scores). Significant differences were also found for IES avoidance median scores between onset and middle confinement (*p* = 0.002), but not for IES intrusive symptoms median scores (*p* = 0.062). For the male group, significant differences were found between onset and ending both for both IES intrusive and avoidance symptoms median scores (*p* < 0.001 and *p* = 0.024, respectively). The other significant differences were between onset and middle confinement (for intrusive symptoms median scores, *p* = 0.002; but not for avoidance symptoms median scores, *p* = 0.904), as well as between middle and ending confinement (for avoidance symptoms median scores, *p* = 0.018; but not intrusive symptoms median scores, *p* = 0.428).

**Table 4:** Within-group comparisons by gender of the psychological measures.

	**Female group (*****n*** **= 123)**							**Male group (*****n*** **= 41)**						
	**Onset (March 24–April 6)**	**Middle (April 7–April 20)**	**Ending (April 21–May 4)**					**Onset (March 24–April 6)**	**Middle (April 7–April 20)**	**Ending (April 21–May 4)**				
**PANAS**				**χ^2^**	***p***	**W**	***Post-hoc^**a**^***				**χ^2^**	***p***	**W**	***Post-hoc***
Positive affect	28.2 (8.21)	29.3 (9.83)	29.8 (10.0)	0.63	0.731			27.9 (7.51)	27.9 (7.90)	28.4 (8.93)	0.48	0.786		
Negative affect	18.7 (7.14)	17.2 (7.00)	16.7 (7.85)	23.9	<0.001	0.78	O>M>E	15.9 (5.79)	16.5 (8.18)	16.3 (8.16)	0.92	0.631		
**MHI5**														
Total score	64.9 (17.3)	67.2 (18.4)	68.1 (20.3)	4.76	0.092		O < E	74.3 (14.5)	75.7 (16.0)	76.9 (16.6)	3.15	0.208		
**DASS-21**														
Depressive symptoms	3.42 (3.93)	3.44 (4.28)	3.14 (4.12)	2.53	0.283			1.85 (2.72)	2.59 (3.46)	2.54 (3.47)	3.60	0.166		
Anxiety symptoms	2.61 (3.90)	2.18 (3.66)	2.33 (4.02)	3.97	0.137			1.49 (2.44)	1.46 (3.21)	1.46 (2.84)	0.72	0.697		
General stress symptoms	6.15 (4.87)	5.77 (5.03)	5.42 (5.48)	4.25	0.119		O>E	3.00 (3.25)	4.10 (4.16)	3.81 (4.12)	2.07	0.356		
**IES**														
Intrusive symptoms	11.3 (7.25)	9.40 (6.71)	7.94 (7.71)	35.7	<0.001	0.74	O,M>E	9.46 (6.87)	6.56 (6.79)	6.49 (7.60)	17.7	<0.001	0.80	O>M,E
Avoidance symptoms	14.0 (8.96)	11.7 (8.62)	9.95 (9.32)	36.7	<0.001	0.76	O>M>E	10.3 (7.27)	9.68 (7.90)	8.05 (8.12)	7.42	0.025	0.75	O>E

*PANAS, The Positive and Negative Affect Schedule; MHI5, Mental Health Inventory; DASS-21, Depression, Anxiety and Stress Scale; IES, Impact of Event Scale; n.a., not applicable; Kendall's W; O, Onset; M, Middle; E, Ending*.

a *Significant differences between groups indicated*.

No significant differences were found in the response percentages related to the qualitative questions that were asked throughout the three interviews (Table 5). For this reason, it was not considered that there could be a significant relationship between the results obtained in the psychological measures in the three surveys, and possible variations over time in the qualitative questions.

**Table 5:** Comparison of response percentages in the qualitative questions in the three surveys.

	**Onset (March 24–April 6)**	**Middle (April 7–April 20)**	**Ending (April 21–May 4)**	**Cochran's Q**	***p***
**Changes in employment situation**^**a**^					
No	128 (81.0%)	129 (81.6%)	130 (81.6%)	0.57	0.751
Yes	30 (19.0%)	29 (18.4%)	28 (17.7%)		
**Do you consider COVID a threat to your health?**					
No	68 (41.5%	63 (38.4%)	63 (38.4%)	1.35	0.509
Yes	96 (58.5%)	101 (61.6%)	101 (61.6%)		
**Have you been diagnosed with COVID**					
No	162 (98.8%)	160 (97.6%)	160 (97.6%)	4.00	0.135
Yes	2 (1.2%)	4 (2.4%)	4 (2.4%)		
**Measures imposed by the government**					
Agree or strongly agree	66 (40.2%)	66 (40.2)	70 (42.7%)	0.53	0.767
Disagree or strongly disagree	98 (59.8%)	98 (59.8%)	94 (57.3%)		

a *The question was: Have you suffered changes in your employment situation resulting from the confinement?*

## Discussion

This is the first longitudinal study describing gender differences in psychological impact of the COVID confinement in Spain. One of the principal results of the present study is that women showed worse symptomatology in the first assessment, but they recovered during the confinement period. Namely, the results suggest that the female group began the confinement with a higher level of negative emotions (especially symptoms of stress and avoidance) than the male group. However, these differences decreased significantly over the first weeks. In the middle of the confinement, the differences between groups had practically disappeared (except for the results on the MHI5 scale, where the differences remained significant with a moderate effect size). In this sense, the group of women had significant differences between the onset and the ending of confinement for negative affect, mental health score, and intrusive and avoidance symptoms. The intragroup differences in the case of men were limited to the IES scale. These results reinforce the previous analyses, showing that the intragroup improvement was more evident in the group of women than in men, which allowed, possibly, that at the end of confinement no significant differences (intergroup) were found between men and women (contrary to the onset of the confinement). These results are in line with previous studies where they reported that being women was a risk factor linked to worse psychological responses during the first stages of COVID-19 lockdown (Justo-Alonso et al., 2020; Wang et al., 2020b). Gender differences in the symptomatology is supported by many epidemiological studies that reported that women are at a higher risk for developing anxiety and/or depression symptoms (Vesga-López et al., 2008; Lim et al., 2018). In addition, the fact that female group reported higher levels of negative emotions are in line with the biopsychosocial model proposed by Chaplin (2015), in which women are supposed to express greater levels of emotions. In the meta-analytic review carried out by Chaplin and Aldao (2013), the authors reported that girls tend to express more negative internalizing emotions, being in line with the punctuations in negative affect. One possible explanation about the tendency of the results is that the female group might have developed more emotion regulation strategies than the male group, leading to an improvement in the results (Nolen-Hoeksema, 2012).

Related to the symptomatology, Spanish results were slightly slower although similar to those found in Asia, highlighting that around the 20% of the sample presented depressive, anxiety and PTSD symptomatology (González-Sanguino et al., 2020; Ozamiz-Etxebarria et al., 2020; Solomou and Constantinidou, 2020). In this sense, these results are in line with the studies that analyzed the symptomatology that appeared in previous pandemic situations (e.g., SARS in 2003 or H1N1 in 2009) where avoidance symptoms, fear, sadness or stress symptomatology were registered in people in confinement (Hawryluck et al., 2004; Wheaton et al., 2012; Brooks et al., 2020).

In addition, we found a significant relationship between age and symptomatology. Younger participants showed higher stress, anxiety, and depressive symptomatology. These results are in line with previous studies (Jiménez et al., 2020; Sandín et al., 2020). Young people showed higher avoidance symptoms that can be associated with increases in post-traumatic stress symptoms. Previous studies have related an increase in PTSD during COVID confinement and after similar events, such as Middle East Respiratory Syndrome (Jeong et al., 2016; Jiménez et al., 2020).

## Limitations

The current study has several limitations. First, employing snowball sampling through social media implies that the sample cannot be considered representative of the Spanish general population. Online tools limit access to persons who are not used to this technology, such as the elderly population. Second, the use of self-reported measures is a limitation shared with previous studies worldwide carried out during the first stages of the pandemic (Justo-Alonso et al., 2020; Liu et al., 2020; Tull et al., 2020; Wang et al., 2020b). Third, the sample size is not large enough to draw definitive conclusions. Therefore, future research should include representative samples of the general Spanish population, in the event of a recurrence of the pandemic situation.

## Conclusions

The results of the studies carried out during the COVID confinement highlight the need for developing strategies to reduce the psychological impact of this global situation. In fact, the current unprecedented worldwide situation, the long-term psychological consequences are unknown and there is a need for global actions in order to promote the well-being of the populations. Following the proposal by Wang et al. (2020a), there is a need for online mental health training for the professionals. Two metanalysis carried out before the COVID showed that online psychological interventions showed effect in reducing depressive symptoms in non-depressed population (Rigabert et al., 2020) and in reducing symptoms of anxiety, distress and depression in chronic health populations (White et al., 2020). In this sense, online psychological treatments should be improved in order to respond the need for treatment after confinement periods.

## Data Availability Statement

The raw data supporting the conclusions of this article will be made available by the authors, without undue reservation.

## Ethics Statement

The studies involving human participants were reviewed and approved by Universidad Loyola Andalucía. The patients/participants provided their written informed consent to participate in this study.

## Author Contributions

JF-C prepared the data sets and was in charge of the data analysis. AR-G, DMR, and ÓJ described the theoretical framework and were in charge of the literature research. AR-G participated in the data collection. All authors participated in the selection of the instruments for the assessment and participated in this manuscript.

## Conflict of Interest

The authors declare that the research was conducted in the absence of any commercial or financial relationships that could be construed as a potential conflict of interest.
